# Supplementary material for: Causal Relationship between Aging and Anorexia Nervosa: A White-Matter-Microstructure-Mediated Mendelian Randomization Analysis
Source: Biomedicines. 2024 Aug 16;12(8):1874. doi: 10.3390/biomedicines12081874 (PMC11351342; doi:10.3390/biomedicines12081874)
Supplement: Supplementary file 1 [file biomedicines-12-01874-s001.zip › Supplementary Figure/Figure S8/Leaveoneout plot of MR analysis for TL to ubm-b-1457.pdf]

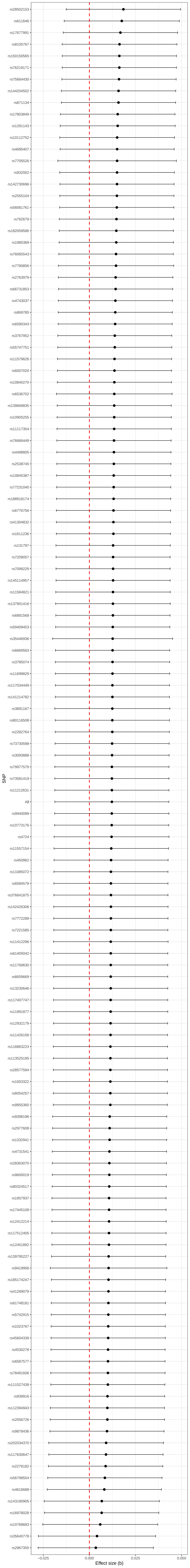

rs28502153
rs611646
rs17677991
rs8105767
rs150150565
rs76219171
rs75664430
rs144204502
rs871134
rs17803849
rs1291143
rs10112752
rs4695407
rs7705526
rs932002
rs142730696
rs2555104
rs56061761
rs762679
rs182059586
rs1985369
rs76065543
rs7790856
rs2763979
rs66731853
rs4743037
rs869785
rs6590343
rs3767952
rs55747751
rs11579626
rs6007020
rs10840270
rs6536702
rs139669835
rs10905255
rs11117354
rs76666449
rs4498805
rs2538745
rs10845387
rs77231040
rs188918174
rs6776756
rs41304832
rs1611236
rs131797
rs7209057
rs7099229
rs145114957
rs11584821
rs137901416
rs6881568
rs59409453
rs35446936
rs6669563
rs3785074
rs11699829
rs117034449
rs141214782
rs3891167
rs80116508
rs2282764
rs73730598
rs3093888
rs79977579
rs73581419
rs11212631
All
rs9940099
rs10773176
rs4724
rs11557154
rs450962
rs11085072
rs6584579
rs376641875
rs142426306
rs7772289
rs7221585
rs11412296
rs61405042
rs11769630
rs6659669
rs13230646
rs117407747
rs11991877
rs12932179
rs11426156
rs116863223
rs113525195
rs28577594
rs1003322
rs6054257
rs9955360
rs9398196
rs2977608
rs1332941
rs4731541
rs28363070
rs9600019
rs80324517
rs1957937
rs17445108
rs12412214
rs117512405
rs12451892
rs139795227
rs9419958
rs185174247
rs41269079
rs61748181
rs5742915
rs1023767
rs45604339
rs4530278
rs6587577
rs78491606
rs111527438
rs939916
rs112394943
rs2056726
rs9878436
rs202034370
rs117630647
rs2276182
rs56799554
rs4616688
rs143190905
rs16978028
rs10768683
rs35640778
rs2967355
SNP
-0.025
0.000
0.025
0.050
Effect size (b)
